# Supplementary material for: Digital Microbe: a genome-informed data integration framework for team science on emerging model organisms
Source: Sci Data. 2024 Sep 4;11:967. doi: 10.1038/s41597-024-03778-z (PMC11374999; doi:10.1038/s41597-024-03778-z)
Supplement: Supplementary file 1 — Supplementary Information [file 41597_2024_3778_MOESM1_ESM.docx]

**Supplementary Information** for “*Digital Microbe, a genome-informed data integration framework for team science on emerging model organisms”*

Iva Veseli^1,2,‡^, Michelle A. DeMers^3,‡^, Zachary S. Cooper^4,‡^, Matthew S. Schechter^5^, Samuel Miller^6^, Laura Weber^7^, Christa B. Smith^4^, Lidimarie T. Rodriguez^8^, William F. Schroer^4^, Matthew R. McIlvin^7^, Paloma Z. Lopez^7^, Makoto Saito^7^, Sonya Dyhrman^9^, A. Murat Eren^1,2,6,10,11,†^, Mary Ann Moran^4,†^, Rogier Braakman^3,†^

^1^Helmholtz Institute for Functional Marine Biodiversity, 26129, Oldenburg, Germany

^2^Alfred Wegener Institute Helmholtz Centre for Polar and Marine Research, 27570, Bremerhaven, Germany

^3^Department of Earth, Atmospheric, and Planetary Sciences, Massachusetts Institute of Technology, Cambridge, MA 02139, USA

^4^Department of Marine Sciences, University of Georgia, Athens, GA 30602, USA

^5^Committee on Microbiology, The University of Chicago, Chicago, IL 60637, USA

^6^Bay Paul Center, Marine Biological Laboratory, Woods Hole, MA 02543, USA

^7^Woods Hole Oceanographic Institution, Falmouth, MA 02543, USA

^8^Department of Microbiology and Cell Science, University of Florida, Gainesville, FL 32611-0180, USA

^9^Lamont-Doherty Earth Observatory, and the Department of Earth and Environmental Sciences, Columbia University, New York, NY 10032, USA

^10^Institute for Chemistry and Biology of the Marine Environment, University of Oldenburg, Oldenburg, Germany

^11^Marine ‘Omics Bridging Group, Max Planck Institute for Marine Microbiology, 28359 Bremen, Germany

† Corresponding authors: meren@mbl.edu; mmoran@uga.edu; [braakman@mit.edu](mailto:braakman@mit.edu)

‡ These authors contributed equally

# Supplementary Figures


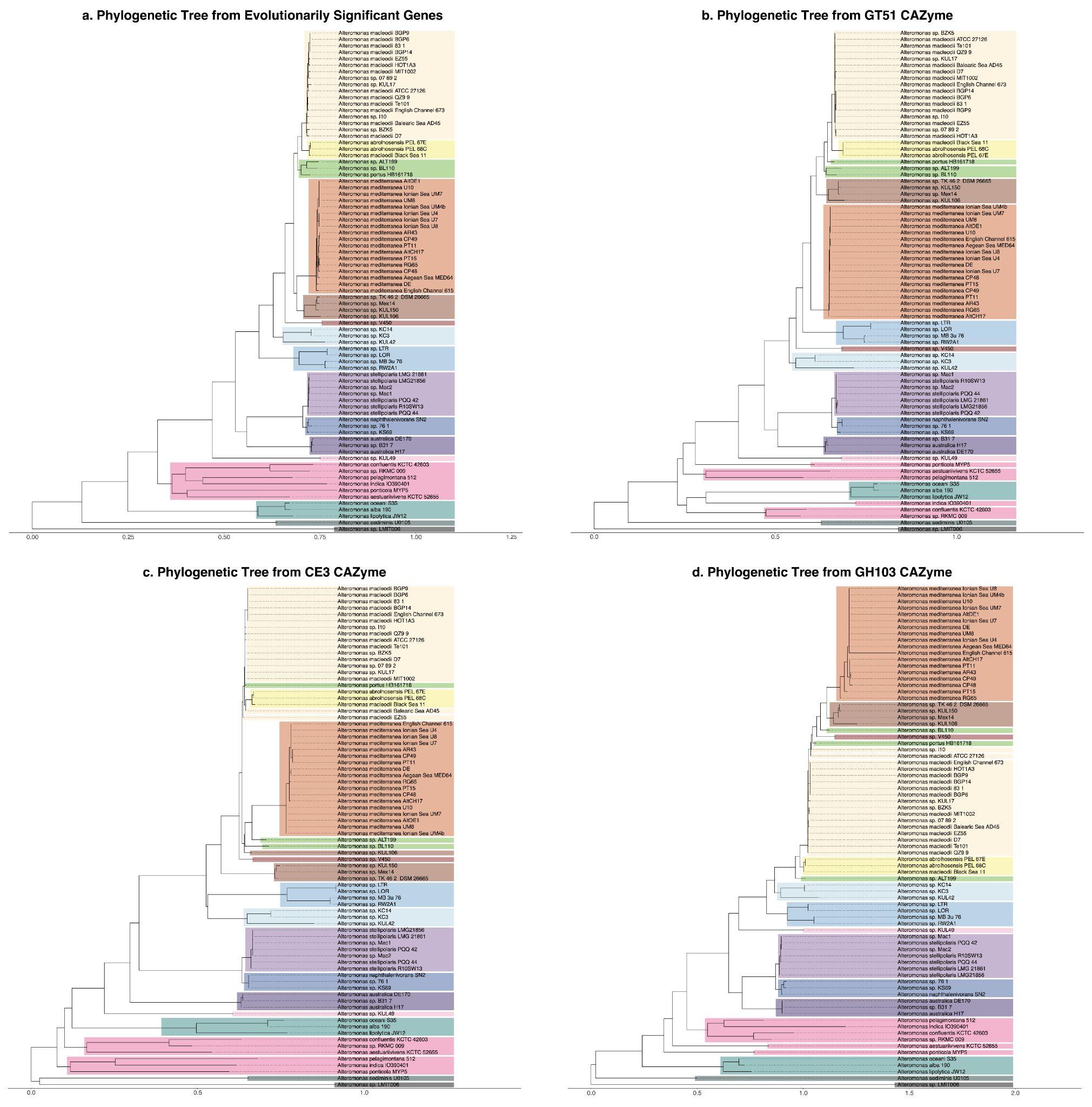


**Supplementary Figure 1. Individual phylogenies of core genes (a) and CAZymes (b, c, d) from the *Alteromonas* pangenome.** The core genes (n=111) used to build the accepted phylogeny in (a) consist of those found in all 78 isolate genomes as single copy genes (‘--max-num-genes-from-each-genome’ set to 1), with the ‘--max-functional-homogeneity-index’ set to 0.9 and ‘--min-geometric-homogeneity-index’ set to 0.925. The CAZyme phylogenies (b, c, d) are each built from the alignment of one gene - GT51, CE3, and GH103, respectively. Clades are highlighted based on color assignments in Supplementary Figure 2. The horizontal axis provides a means for interpreting branch length as a measure of genetic change.


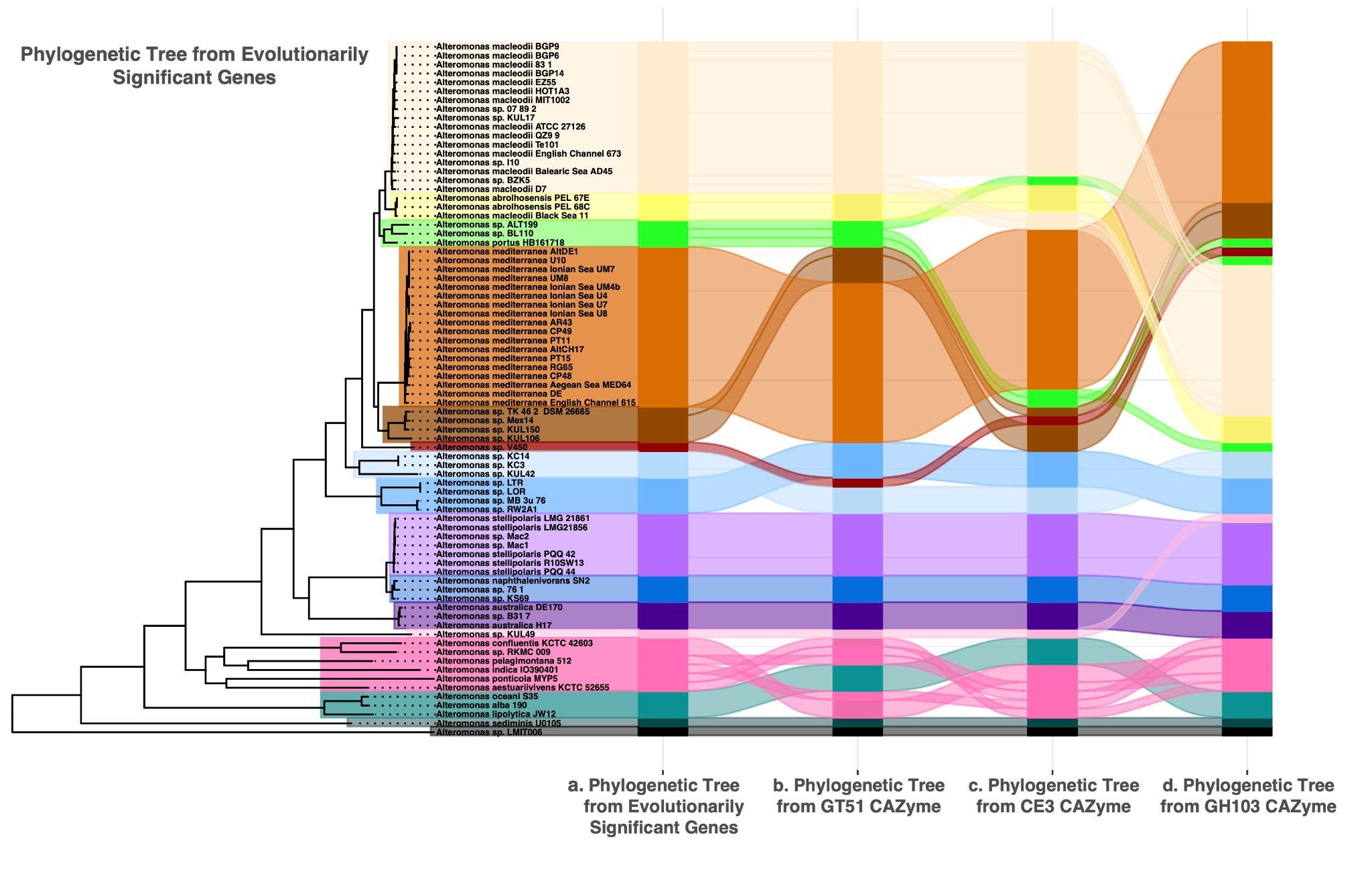


**Supplementary Figure 2. Comparison of core gene (a) and CAZyme (b, c, d) phylogenies for the *Alteromonas* pangenome.** The core genes (n=111) used to build the accepted phylogeny in (a) consist of those found in all 78 isolate genomes as single copy genes (‘--max-num-genes-from-each-genome’ set to 1), with the ‘--max-functional-homogeneity-index’ set to 0.9 and ‘--min-geometric-homogeneity-index’ set to 0.925. The CAZyme phylogenies (b, c, d) are built from the alignment of one gene each. The alluvial plot displays the movement of clades within the tree. Further branch definition of each tree is shown in Supplementary Figure 1.

# Supplementary Tables

**Table S1.** Transcriptome samples included in the *Ruegeria pomeroyi* Digital Microbe.
